# Effects of temperature and precipitation changes on shifts in breeding phenology of an endangered toad

**DOI:** 10.1038/s41598-023-40568-w

**Published:** 2023-09-04

**Authors:** Andrea Dalpasso, Daniele Seglie, Paolo Eusebio Bergò, Andrea Ciracì, Mariachiara Compostella, Lorenzo Laddaga, Milo Manica, Gaia Marino, Irene Pandolfo, Giovanni Soldato, Mattia Falaschi

**Affiliations:** 1 Department of Biology, Université Laval, 1045 Avenue de la Médecine, Québec, G1V 0A6 Canada; 2 Department of Environmental Science and Policy, Università degli Studi di Milano, Via Celoria 10, 20133 Milan, Italy; 3 Eleade, C.le Montresco 1, 10010 Chiaverano, TO Italy; 4 Department of Life Sciences and Systems Biology, Università degli Studi di Torino, Via Accademia Albertina 13, 10123 Turin, Italy; 5 Società di Scienze Naturali del Verbano Cusio Ossola, Museo di Scienze Naturali, Collegio Mellerio Rosmini, 28845 Domodossola, Italy; 6 Parco Lombardo della valle del Ticino, Via Isonzo 1, 20013 Pontevecchio di Magenta, MI Italy; 7 Department of Chemical Science, Life and Environmental Sustainability, Università degli Studi di Parma, Parco Area delle Scienze 11/A, 43124 Parma, Italy

**Keywords:** Climate-change ecology, Wetlands ecology

## Abstract

In the last century, a plethora of species have shown rapid phenological changes in response to climate change. Among animals, amphibians exhibit some of the greatest responses since their activity strongly depends on temperature and rainfall regimes. These shifts in phenology can have negative consequences for amphibian fitness. Thus, understanding phenological changes in amphibians is pivotal to design conservation actions to mitigate climate change effects. We used data on Common Spadefoot Toad (*Pelobates fuscus*) reproductive migration to wetlands over a period of 8 years in Italy to (i) identify the factors related to breeding migrations, (ii) assess potential phenological shifts in the breeding period, and (iii) determine which climatic factors are related to the observed phenological shifts. Our results showed that toads migrate to spawning sites preferably in early spring, on rainy days with temperatures of 9–14 °C, and with high humidity. Furthermore, despite an increase in average temperature across the study period, we observed a delay in the start of breeding migrations of 12.4 days over 8 years. This counterintuitive pattern was the result of a succession of hot and dry years that occurred in the study area, highlighting that for ephemeral pond breeders, precipitation could have a larger impact than temperature on phenology. Our results belie the strong presumption that climate change will shift amphibian phenology toward an earlier breeding migration and underline the importance of closely investigating the environmental factors related to species phenology.

## Introduction

Phenology refers to the timing of cyclical events (e.g. spring flowering or seasonal migrations) in relation to a series of biotic and abiotic elements. It is usually determined by a combination of endogenous and environmental components^1,2^ and plays a central role in the life cycle of a multitude of organisms^3–5^. The timing of breeding is a characteristic that has important implications for fitness in many animals^6,7^.

In the last decades, the environmental factors related to phenological patterns have gained interest due to climate change^8^. Climatic changes can shift the phenology of species, potentially leading to phenological mismatch, that is the mismatch between demand and availability of natural resources^9–11^. Climate-driven shifts in phenology are likely to have a deep impact at population and community levels^12^, as phenological mismatch can alter resource availability and trophic interactions^13,14^. This can affect demography by reducing reproductive output, with diminished recruitment rates, and ultimately lead to a decline in population size, which can possibly influence the risk of extinction^13,15,16^. There is a fundamental need, therefore, to recognize and quantify the leading causes of phenological shifts and analyse the responses of the species to a changing environment.

Phenology has a key role in amphibian lives, chiefly in species living in temperate regions where many activity cycles are determined by seasonality^17,18^. For instance, climatic features such as temperature and precipitation play a central role in determining breeding migrations and changes in phenology^19^, especially for species that rely on ephemeral waterbodies for reproduction and larval development^20,21^. Adults must use environmental cues to time their movements with seasonal precipitation events that fill wetlands to allow larvae to have enough time to develop before water levels recede. Furthermore, adults must time above-ground movements with meteorological conditions to avoid desiccation risk. Breeding phenology can also vary among individuals from the same population due to several factors, such as sex and size^18, 22^. Indeed, in many species males migrate earlier than females to the breeding sites, in order to compete for the best spots in the pond where they will wait for the females^23^. Studies on amphibian responses to climate change have shown variability in phenological shifts across species and populations, with both earlier spring breeding^18,24–28^ and delays in seasonal migrations^29,30^. This means that the direction, timing, and strength of these changes can depend on specific environmental conditions experienced at the local scale, along with other important factors such as endogenous rhythms^19,31–34^. Shifts in amphibian phenology often have negative consequences for their fitness, as phenological mismatch can alter habitat availability and predator–prey dynamics, and consequently compromise community stability^9,14,17,25,35^. Indeed, even if habitat loss, diseases, and pollution are seen as the major threats to amphibian's persistence^36^, phenological changes can have a non-negligible effect on survival, leading to the decline and extinction of species that are less resistant to climate change^37–41^. Thus, it is very important to understand how climatic changes will affect amphibian phenology, in order to design adequate conservation and management actions.

Here, we used data on Common Spadefoot Toad (*Pelobates fuscus*) breeding migration to wetlands over a period of 8 years in NW Italy (Supplementary Fig. S1) to evaluate the status of the population and especially to assess which factors predict variations in toad migratory activity over the years. The Italian populations of this species have a great conservation value (listed in Annex II of the Habitats Directive 92/43/EEC) due to the species rarity in the area and the large number of exclusive haplotypes observed^42^. The species status has rapidly deteriorated due to anthropic alteration (i.e. habitat degradation and fragmentation) but it is unclear what additional threat climate change may pose. Understanding the consequences of climate change requires knowledge of the potential impact of phenological changes, however, no studies so far have analysed possible shifts in the breeding phenology of the Common Spadefoot Toad, which may have important effects on its reproductive success, potentially undermining its long-term persistence. More specifically, our goal was to (i) identify the factors related to the breeding migration of the Common Spadefoot Toad, (ii) assess potential phenological shifts in the breeding phenology of this species over the last decade, and (iii) determine which climatic factors are related to the observed phenological shifts. To do that, we (i) evaluated the effect of different variables (date, temperature, precipitation, humidity, wind, and moon phase) on the activity patterns of toads moving to their spawning sites. Climate change can alter breeding phenology in different ways. We may observe either a shift of the whole breeding period or a shortening of its duration through a shift in the beginning or the end of breeding activities^4,43^. Hence, we (ii) evaluated if the beginning, the peak, and the end of the migration shifted over time, and (iii) assessed if the observed phenological shift was related to climatic changes and if it had an impact on population abundance over the years.

## Results

### Factors related to breeding phenology

**Figure 1:**
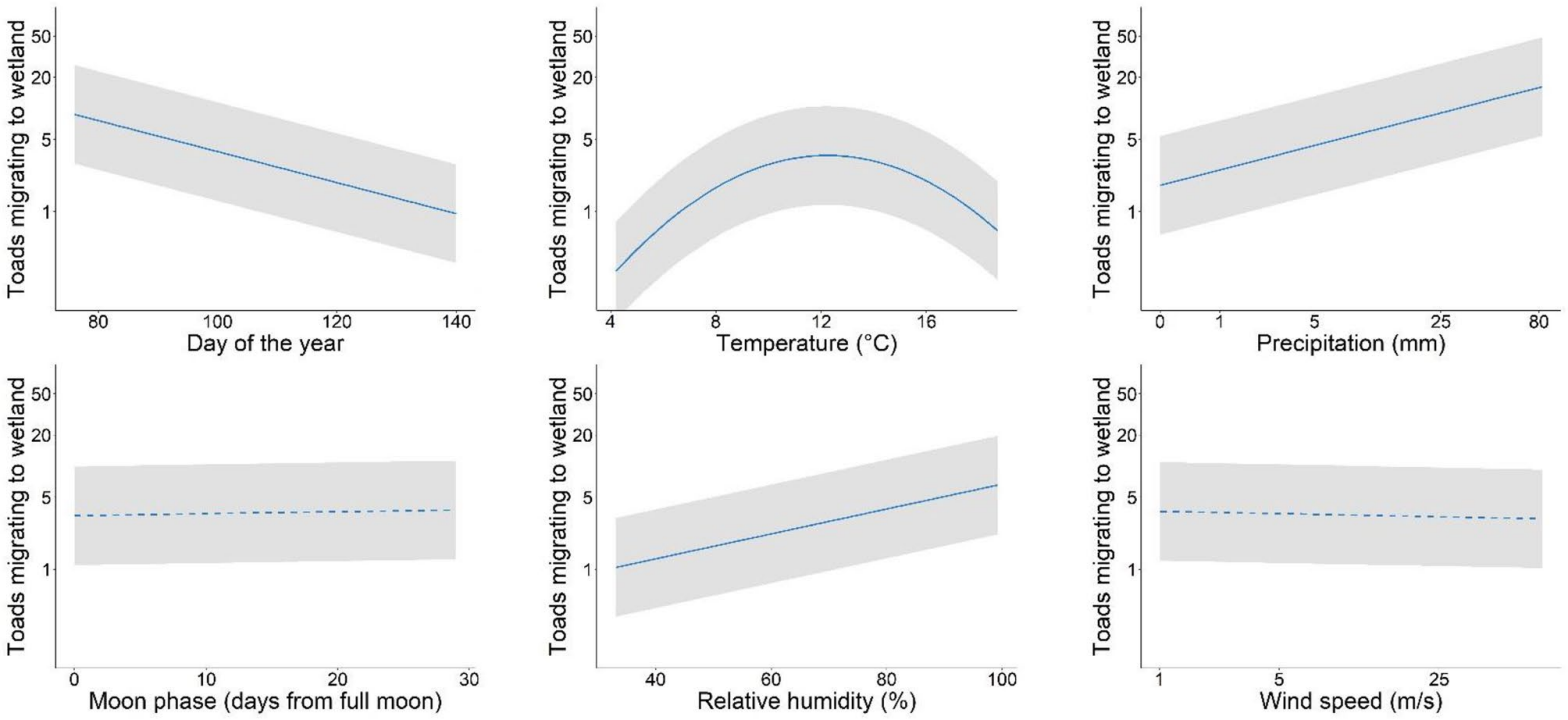
Effects of environmental factors on the number of Common Spadefoot Toad daily migrating to breeding wetlands. The continuous line represents the mean estimate (dashed: not significant; continuous: significant), while the grey area represents the 95% confidence interval.

The daily number of toads that migrate to the spawning sites was correlated both to the date and to several environmental factors (Fig. 1, Table 1, Supplementary Fig. S2). The model showed a negative and significant effect of the day of the year (β = − 0.50, *p* < 0.001), a positive and significant effect of the precipitation amount (β = 0.85, *p* < 0.001) and of the relative humidity (β = 0.39, *p* = 0.002), and a quadratic and significant effect of the mean temperature (quadratic coefficient β = − 0.34, *p* < 0.001) on the breeding phenology of the species, meaning that a higher number of toads migrate at the beginning of the reproductive season, on rainy and humid nights with temperatures between 9 and 14 °C (Fig. 1, Table 1). Moon phase and wind speed showed no significant relationship with the number of toads migrating to wetlands (Table 1). Two alternative models ran with minimum and maximum temperatures instead of mean temperature, showed highly consistent results but lower performance (Supplementary Tables S1 and S2). When individuals were divided by sex, we found no difference between males and females (Supplementary Table S3).

**Table 1:** Parameters estimated by the model analysing the factors related to the number of Common Spadefoot Toads migrating to breeding wetlands each day. Statistically significant parameters are highlighted in bold. se: standard error. The superscripts “2” indicate quadratic terms.

	Coefficient ± SE	*z*-value	*p*-value
Intercept	1.34 ± 0.55	2.43	0.01
Date	**− 0.5 ± 0.1**	**− 4.93**	** < 0.001**
Cumulative daily precipitation	**0.85 ± 0.09**	**9.41**	** < 0.001**
Daily mean temperature	**− **0.12 ± 0.11	**− **1.1	0.27
Daily mean temperature^2^	**− 0.34 ± 0.06**	**− 5.87**	** < 0.001**
Relative humidity	**0.39 ± 0.13**	**3.08**	**0.002**
Wind speed	**− **0.02 ± 0.09	**− **0.27	0.79
Moon phase	0.03 ± 0.03	0.95	0.34
Moon phase^2^	**− **0.0009 ± 0.001	**− **0.82	0.41

### Shift in breeding phenology

Over the study period, we found a positive and significant shift in the beginning of migration (measured as the 10th percentile of toads migrating to the wetlands in each year) (β = 1.55, *p* < 0.001), meaning that the species is postponing the beginning of the breeding migration to the wetlands by approximately 2 days each year. This resulted in an average shift of the breeding migration of 12.4 days from 2013 to 2021 (Table 2; Supplementary Fig. S3).

**Table 2:** Parameters estimated by the three models assessing changes in breeding phenology of the Common Spadefoot Toad between 2013 and 2021. The three tables show shifts in a) beginning, b) peak, and c) end of breeding migrations. Statistically significant shifts are highlighted in bold. se: standard error.

	Coefficient ± SE	*z*-value	*p*-value
(a) Beginning of breeding migrations (10th percentile)			
Intercept	**− **88.3 ± 0.85	**− **104.38	< 0.001
Year	**1.55 ± 0.35**	**4.48**	** < 0.001**
(b) Peak of breeding migrations (50th percentile)			
Intercept	110.4 ± 3.87	28.54	< 0.001
Year	**− **0.66 ± 1.58	**− **0.42	0.674
(c) End of breeding migrations (90th percentile)			
Intercept	118.41 ± 3.45	34.30	< 0.001
Year	**− **0.13 ± 1.41	**− **0.09	0.926

Conversely, the peak and end of breeding migrations (50th and the 90th percentile of migrating toads) did not change significantly over the same period (*p* = 0.67 for the 50th percentile and *p* = 0.92 for the 90th percentile), meaning that the peak and the end of the breeding migration were not postponed like the start of the migration (Table 2; Supplementary Fig. S3).

### Factors related to phenological shift

**Figure 2:**
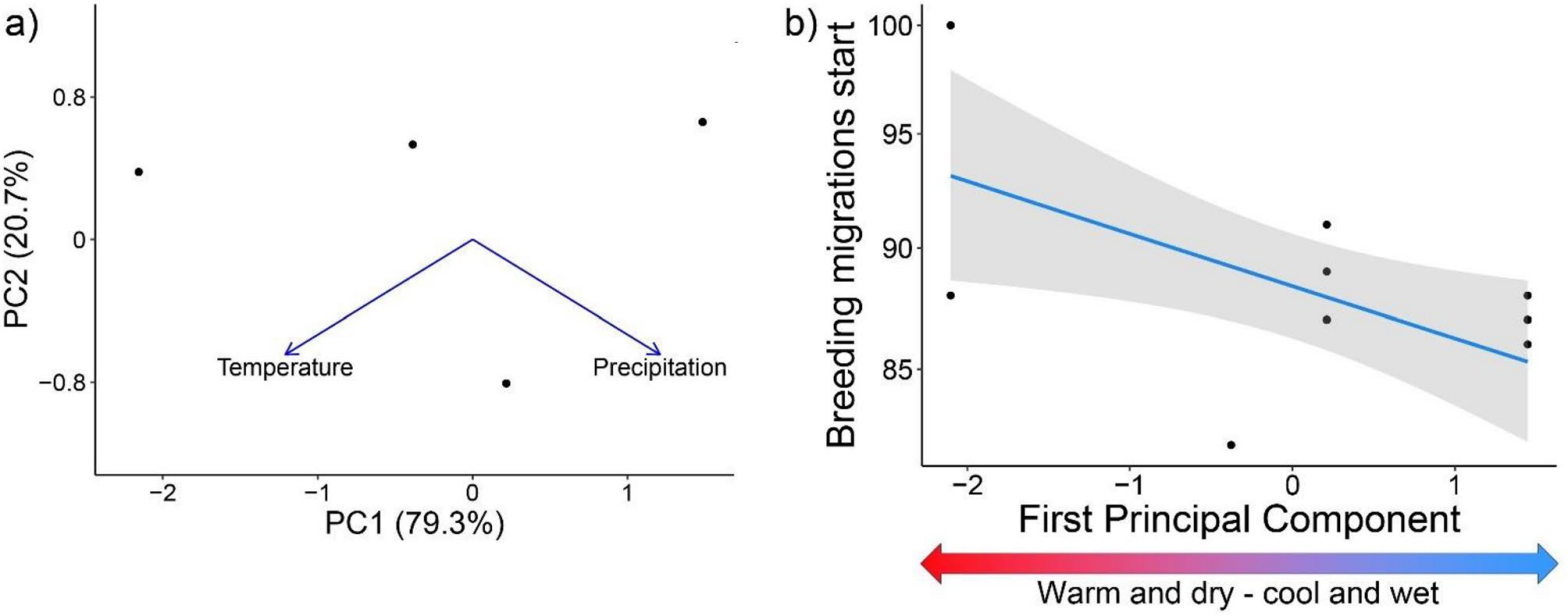
Relationships between climatic factors and the day of the beginning of migration. Panel (**a**) shows the result of the principal component (PC) analysis including temperature and precipitation in the study area over the sampling years. Low values of PC1 described dry and hot years, while high values of PC1 indicated rainy and cool years. Panel (**b**) shows the relationship between climate (PC1) and the day of the start of migration. Points represent the day of migration ad different sites in different years, the blue line represents the mean estimate, and the grey area represents the 95% confidence interval.

**Figure 3:**
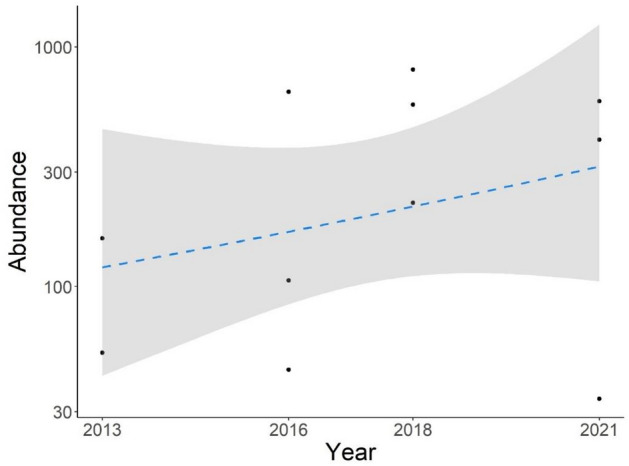
Estimated trend of total abundance of the Common Spadefoot Toad in the study area. Points represent the average estimates for each site for each year, the dashed line represents the mean estimate and the grey area represents the 95% confidence interval.

To test if temperature and precipitation of the period preceding the breeding migration influenced the observed phenological shift, we first performed a principal component analysis (PCA) to get uncorrelated variables and avoid model overfitting. The first PC described nearly 80% of the variation in the data, therefore we kept only the scores of this PC as explanatory variable in the subsequent model. Low values of PC1 described dry and hot years, while high values of PC1 indicated rainy and cool years (Fig. 2a). The model showed a negative and significant relationship between the PC1 and the day of beginning (10th percentile) of migrating individuals (β = − 2.32 ± 0.87, *p* = 0.008), indicating that in years when the period preceding the breeding season was warmer and drier, the start of the breeding migration was postponed (Fig. 2b). Despite the observed shift in breeding phenology, population abundance was not affected (Fig. 3). Indeed, the abundance model showed no significant changes in the study area over time (β = 0.15 ± 0.1, *p* = 0.16), with the number of adult toads remaining stable between 100 and 300 individuals (Fig. 3).

## Discussion

Our study analysed different aspects of the Common Spadefoot Toad population living in the Site of Community Importance “Paludi di Arsago”. We first showed that breeding migrations are related to the day of the year, daily cumulative precipitation, daily humidity levels, and daily mean temperature. Furthermore, we showed that the onset of breeding migration was delayed by 12.4 days over 8 years. Such phenological shift was related to changes in temperature and precipitation regimes, but the studied population did not show significant changes in abundance.

The daily number of Common Spadefoot Toads migrating to breeding wetlands was jointly determined by date, temperature, humidity, and the cumulative precipitation (Table 1). The number of toads migrating to the wetlands was negatively correlated with the date, meaning that most individuals moved to the reproductive sites at the beginning of the breeding season (usually by the end of March). The synchronous arrival of males and females at the start of the breeding season appears to be a common feature for ephemeral pond breeders that expect a high probability of reproductive failure^44,45^. Indeed, as reproduction takes place only once a year for explosive breeders, the timing of the arrival at the breeding sites is fundamental to ensure high reproductive success and full larval development^21^.

Amphibians use multiple environmental cues to time breeding activity, and for many temperate species the onset of the migration occurs on rainy days and/or when temperature rises above species-specific thresholds^46^. We found a strong positive correlation between daily relative humidity, daily cumulative precipitation, and the number of migrants, meaning that toads showed a great propensity for movement just following or during precipitation events. These results are consistent with the literature knowledge of the species, where peaks of migratory activity match with rainy days^47,48^. This is not surprising, considering that most amphibians have only limited desiccation tolerance, and therefore their activity will be higher under wet conditions^30^. Furthermore, precipitation and soil moisture can be used by the species as cues to assess the water levels since the timing of ephemeral pond-filling is usually determined by rainfall events. Breeding shortly after pond-filling is essential to maximize tadpoles' development and to minimize competition and predation^49^.

As expected, temperature was another important factor related to the species’ activity in spring, as increasing temperatures are often one of the initial cues triggering the onset of the migration^4,50^. Indeed, we found a quadratic relationship between the number of migrants and the daily mean temperature, meaning that toads mainly move during days with temperatures ranging between 9 and 14 °C. Being ectotherms, an increase in external temperature is likely to result in increased metabolic activity, thus increasing the likelihood of triggering breeding migration^50^. In fact, inputs for the start of the breeding migration are likely provided by hormonal changes (e.g. prolactin), which normally are themselves triggered by temperature changes and photoperiod^51^. At the same time, moving at too hot external temperatures can be dangerous for amphibians as they can increase the risk of death by desiccation^21^.

The effect of moon on amphibian breeding phenology has been widely recognized^31,52^, however, we did not find any effect of the moon phase on the breeding migration of the Common Spadefoot Toad (Table 1). Costs and benefits to amphibians migrating under full/new moons are highly species-specific and are related to the ecology of the species^52^; our results suggest that other factors (e.g., temperature, precipitation, humidity) have a greater influence on triggering the onset of toad migrations. Indeed, for ephemeral pond breeders that have a short time window for reproduction and larval development, the migration should be more associated with events that increase the successful development of larvae, such as precipitation events that raise water levels^44^.

So far, the existing literature has documented a general trend of anticipating the breeding phenology across taxonomic groups in response to climate change^10,53–55^. Among animals, amphibians are the group that displays the strongest relationship between phenological shifts and changing climate, with an average spring migration advancement that goes from 2.6 to 13 days, depending on the species^24,26,28,56–59^. Even if phenological delays have been reported in some amphibians, most of them were attributed to fall-breeding species where delay in migration is expected due to warmer summers^30^. Our study is one of the very few to find a delay in the reproductive migration of a spring-breeding amphibian (12.4 days in 8 years), even though there has been an increase in the average temperature over the years as in other studies (~ 0.4 °C each year). Moreover, the magnitude of the delay was considerably larger than the only other study known to us in which a delay in spring breeding migration was documented (2.8 days in 20 years^29^).

We found that the delay in breeding migrations is correlated to changes in the climatic conditions, namely the occurrence of more hot and dry springs over the years (Fig. 2). The relative importance of temperatures and precipitation in modifying phenological patterns of activities is highly influenced by taxonomical bias^19^, with species reacting differently to climatic changes even if they live in the same area^17,60^. Many studies on amphibian breeding phenology focused on very restricted taxonomic groups (e.g. *Rana* and *Bufo* genera are overrepresented) which are known to usually reproduce in permanent or semi-permanent waterbodies^23^. In these cases, increasing temperatures played a major role in determining phenological advancements, with precipitation having little or no effect^61–64^. Conversely, for ephemeral pond breeders like the Common Spadefoot Toad, precipitation is likely to be the main predictor of phenological changes. Indeed, for these species, hydroperiod length determines breeding success, and short hydroperiods caused by dry years can lead to complete reproductive failure^21^, leading not only to phenological mismatch but also to population decline and extinctions^37,39^.

Interestingly, our study contrasts with the results of the only other study that analysed changes in the reproductive phenology of the Common Spadefoot Toad. In fact, Yermokhin et al. ^65^ observed that populations of the Medveditsa River floodplain (Saratov Oblast, Russia) anticipated the spring spawning migration by approximately 8/9 days in 100 years. This was caused by a shortening of the winter hibernation due to increased soil temperature, which bring the toads to an earlier migration to the spawning lakes. Species with large home ranges often show geographical variation among populations^64^, and these differences are likely triggered by both genetic and environmental constraints. Local adaptation and phenotypic plasticity of each population can then determine its vulnerability to environmental changes. Our work underlines the importance of studying the factors related to phenological shifts in different populations, especially for those that may live in suboptimal environmental conditions such as range margins, which therefore could be more susceptible to environmental changes.

Temporary ponds are critical spawning habitats for many amphibians, as here breeding can occur in the absence of voracious predators like fishes^66^. At the same time, temporary pond breeders can pay the cost of a narrower larval development time due to a much shorter hydroperiod. Global climate change is predicted to increase the frequency of drought, with ephemeral ponds experiencing increasingly shorter hydroperiods^67^. Changes in hydroperiod can be an important environmental stressor for amphibian larvae^68^, and it can significantly decrease species fitness through a reduction in survival rates before or after metamorphosis^69^. Therefore, shorter hydroperiods can lead to lower recruitment rates and eventually to reproductive failure^21^, and a succession of dry years can bring populations to decline and ultimately to their extinction^39,60^.

Another consequence of phenological shifts can be the alteration of interspecific interactions such as competition and predator–prey dynamics^14^. In our study area, the phenological delay of the Common Spadefoot Toad is causing a greater temporal overlap with the breeding activity of other amphibians (such as the Italian Crested Newt *Triturus carnifex* and the Edible Frog *Pelophylax kl. esculentus*), which can act as competitors and/or predators of tadpoles and froglets (Supplementary Fig. S4). Although our model showed no significant changes in species abundance (Fig. 3), decreases in population dimensions may occur in the future because of  lower recruitment due to an increase in predation and/or competition pressures. Larval anurans mainly compete for limited food resources, where one species may outcompete another one because of its better ability to find and consume resources, thereby reducing the food supply for others^70^. The Edible Frog is a generalist and well-adaptable species, and it has a population size significantly larger than the one of the Common Spadefoot Toad in our study area. Therefore, Edible Frog tadpoles are likely to consume significantly more resources, increasing competitive pressure on the Common Spadefoot Toad. In fact, it has been proved that common species can impact the population dynamics of rarer species, due to the effect of exploitative competition^70^. Furthermore, direct predation of froglets by adult Edible Frogs has been observed in our study area (Supplementary Fig. S4), proving that the Edible Frog can also act as a predator for the Common Spadefoot Toad.

Despite the results obtained, our analyses are not exempt from limitations. For instance, when analysing the factors related to phenological shifts, we included data from only 4 years within the 8-years timespan. Therefore, these results should be interpreted very cautiously, since analyses performed on small datasets can be very sensitive to outliers (for example, abnormal hot and dry or wet and cold years;^71^). However, while long-term monitoring programs are essential, they are often complex to maintain, and even limited information can be precious to give directions to conservation planning^72^.

In conclusion, we showed that despite global warming often translates into an advancement of amphibian breeding phenology, some species can display a phenological delay due to the occurrence of drier years. Our study underlines the importance of investigating the factors related to phenology in animals over a wider range of taxa and belies the strong presumption that increasing temperatures will shift the phenology of ectotherms through early migration. Indeed, populations in humid areas where rainfalls are more stable may be more likely to change their phenology in response to temperature, whereas precipitation may be the limiting factor for populations living in dryer areas^19^. The study of breeding phenology is essential to understand how a species will react to the ongoing climate change, as phenological shifts can severely affect population dynamics and therefore the persistence of species. This information will be fundamental for the planning of conservation programs aimed at mitigating the effects of climate change.

## Materials and methods

### Study species

The Common Spadefoot Toad (*Pelobates fuscus*) is a medium-sized fossorial amphibian widespread in Central and Eastern Europe but occurring in Italy only with a few small and isolated populations in the Po Valley. Toads inhabit soft or sandy soils, where they can burrow during winter and re-emerge in spring for reproductive migration^73^. The species is an explosive breeder, with both sexes migrating to the wetlands with the incoming of the first late-winter/early-spring rainfalls. The breeding sites are temporary waterbodies characterized by a marked seasonality^48^; females lay 1500/2000 eggs in a string and the larval period lasts for 2–4 months^47^. According to the Italian IUCN Red Lists, the Italian populations are listed as endangered due to the reduction and alteration of suitable habitats and the increase of intensive agriculture^47,74^.

### Study area and data collection

In four different years between 2013 and 2021 (2013, 2016, 2018, 2021), we monitored six temporary wetlands within the Site of Community Importance “Paludi di Arsago”, a wooded protected area that hosts the largest population of the Common Spadefoot Toad in Italy^48^. The study area is located in the Lombardy region (NW Italy) and has an extension of 5.4 km^2^ (Supplementary Fig. S1). During the reproductive season, a polythene drift fence 60 cm high and burrowed to a depth of 15 cm was placed to surround the perimeter of each study pond. Pitfall traps (plastic buckets) were located along the perimeter of each fence at approximately 10 m intervals, one on both sides. The use of drift fences and pitfall traps is a common sampling technique for amphibian communities^43,75^; bias and limitations of this technique have been discussed^76–79^ but these have little effect on the assessment of migration dates. Pitfall traps were checked twice a day (early morning and night) and all amphibians were released on the side of the barrier opposite to their capture, assuming that animals captured outside the barrier were moving towards the pond and vice versa. For each session, the sex and the total number of individuals trapped were recorded. Since the Common Spadefoot Toad is a nocturnal migrator, we grouped every count of the night session with the ones of the morning session of the following day, considering them as a single migration event. Not all wetlands were monitored in each study year due to two different reasons: two wetlands did not contain water in 3 years and therefore were not monitored; in another site, fishes were introduced in 2018, strongly reducing toad subpopulation, therefore this site was excluded from 2021 monitoring activity. However, these three wetlands hosted overall less than 4% of the total population of Common Spadefoot Toad, therefore we expect that their exclusion in some years did not affect subsequent analyses.

To analyse the phenology of the species, we performed three models based on two distinct datasets (the datasets, including raw counts, and script used to perform the analyses are available at figshare: https://doi.org/10.6084/m9.figshare.23822589).

### Model on the factors related to breeding phenology

We used generalized linear mixed models to assess relationships between the daily number of Common Spadefoot Toads migrating to wetlands and both abiotic and endogenous processes. One of the most well-known endogenous processes is the circadian rhythm, which is strictly related to the light/dark cycle^80^. For this reason, we estimated the number of migrants as a function of the date (expressed as the day of the year). Additionally, we considered several meteorological factors, calculated over the period 7:00–6:00 to include the timespan covered by night and morning toad surveys: minimum, mean, and maximum daily temperatures (both linear and quadratic terms), cumulative daily precipitation, relative humidity, wind speed, moon phase and its quadratic term, and two random effects representing the site and the year of sampling.

Meteorological data were downloaded from the regional agency for the protection of the environment (https://www.arpalombardia.it/). We downloaded daily data of mean, minimum, and maximum temperature, relative humidity, and cumulative precipitation from the meteorological station with the best data quality and closer to our study area (Cavaria con Premezzo municipality; average distance ± SD: 5.6 ± 0.78 km). Wind speed was retrieved from another station located in the Castronno municipality (8 km from the study area). Nocturnal animals have to deal also with a series of environmental conditions that might affect their migration to the breeding sites, such as the lunar cycle^81,82^. Since moonlight can also affect amphibian biology^31, 52, 83^, we also included moon phase data in our analyses. We obtained the dates of full moons from the US Naval Observatory Astronomical Applications Department (https://aa.usno.navy.mil/data/MoonPhases). Then, following Grant et al.^23^, for each of the recorded migration events we calculated the days since the full moon and we assigned to each date a value in the range 0–29, where 0 represents the full moon (Supplementary Fig. S2).

Given the generally high correlation among temperature variables (see Supplementary Table S4) we compared alternative models to select the best-fitting variable. In particular, we ran three alternative models keeping the same independent variables (humidity, precipitation, wind, etc.…) and alternating the temperature variable (i.e. mean, minimum, or maximum temperature). We then used R^2^ and Akaike Information Criterion (AIC) to select the best-performing model. The model including the mean temperature showed both the best R^2^ and AIC (Supplementary Table S2), hence, we present and discuss this model (Fig. 2). Since count data are inherently right-skewed, with a high frequency of days with few migrants and a low frequency of days with a large number of migrants, and considering that nights with zero toads were removed from the analyses in order to avoid issues related to zero-inflation, the model could be fitted with a negative binomial or a quasi-Poisson distribution. Since objective and quantitative standard methods to understand which distribution fit better seem to be lacking^84^, we ran preliminary models comparing the fitting of quasi-Poisson against negative binomial, and we found that negative binomial had a much better fitting (Conditional R^2^ quasi-Poisson: 0.523; Conditional R^2^ negative binomial: 0.793). Therefore, we decided to keep the negative binomial distribution.

### Model on phenological shift in breeding migration

To assess potential phenological shifts in breeding phenology of the Common Spadefoot Toad, we used generalized linear mixed models to analyse if the beginning, peak, and end of migrations changed over the years. Starting from toad daily counts, we calculated three estimates of breeding activity for each year: median, 10th, and 90th percentile arrival dates. The beginning of the migration was estimated as the date when 10% of the toads recorded in a given year had migrated^43^. Similarly, the peak and the end of the migration were estimated using 50% and 90% of the total number of toads recorded. We decided to use the 10th percentile instead of the first arrival date as it is a more reliable indicator of the onset and the end of the breeding migrations, because it eliminates the minor pre- and post-migratory activity that can bias the analyses. We ran three models using the day of the year of the beginning/peak/end of migration as dependent variables, the year as independent variable, and the site as a random effect.

### Model on the factors related to phenological shift

Meteorological conditions preceding the breeding season have been shown to influence the timing of breeding^10,30,64^, hence, we assessed the effects of precipitation and temperature on the changes in toad phenology over time. In so doing, we calculated the mean temperature and the cumulative precipitation of every day of the period previous to breeding migration (beginning of January–first half of March). Preliminary models showed that climatic conditions in the first 2 weeks of March were the ones best explaining the beginning of migratory activity, therefore we used the mean temperature and cumulative precipitation of this period in the following model. Since the two variables showed a non-negligible correlation (Pearson correlation coefficient = − 0.58) and given the restricted number of data available for this analysis, we first performed a principal component analysis (function prcomp, R package stats^85^) to reduce dimensionality and obtain uncorrelated variables. Then, we used the score of the first principal component (PC1) as explanatory variable since it explains an important amount of the variance in the data (79%). In this last generalized linear mixed model, the day of the beginning of breeding migrations was estimated as a function of the PC1 and a site random effect.

### Model on population abundance

To assess potential changes in the population dynamic of the Common Spadefoot Toad in the study area, we evaluated possible variations in toad abundance over the years. Toad abundance was estimated as the total number of toads migrating to the breeding wetlands counted each year in the pitfall traps for each site. We ran a generalized linear mixed model where toad abundance followed a negative binomial distribution, with the year as independent variable and a site random effect. For this analysis, we considered only the three sites that had at least 3 years of data and host almost the entire toad population.

All the analyses were performed in R using the R package glmmTMB^86^. Before running all the analyses, precipitation data were log-transformed and all independent variables were standardized with mean = 0 and variance = 1 to improve convergence and allow the comparison of the effect sizes^87^.

### Ethics statement

All research involving animals was performed in accordance with the national regulations and was conducted under the authorization of National Authorities (Ministero dell’Ambiente della Natura e del Mare; prot. 0,039,992/PNM and 0,063,395/PNM)”.

### Supplementary Information

Supplementary Information.

## Data Availability

All data presented in this article and a script to run the analyses are available at figshare: https://doi.org/10.6084/m9.figshare.23822589.
