# Supplementary material for: Effects of temperature and precipitation changes on shifts in breeding phenology of an endangered toad
Source: Sci Rep. 2023 Sep 4;13:14573. doi: 10.1038/s41598-023-40568-w (PMC10477230; doi:10.1038/s41598-023-40568-w)
Supplement: Supplementary file 1 — Supplementary Information. [file 41598_2023_40568_MOESM1_ESM.pdf]

Supporting Information for:

Effects of temperature and precipitation changes on shifts in breeding phenology of an endangered toad

Andrea Dalpasso, Daniele Seglie, Paolo Eusebio Bergò, Andrea Ciracì, Mariachiara Compostella, Lorenzo Laddaga, Milo Manica, Gaia Marino, Irene Pandolfo, Giovanni Soldato, Mattia Falaschi

Table of contents

- **Figure S1.** Location and satellite view of the study area.
- **Figure S2.** Plot showing the distribution and variation of data used in the model analysing the factors related to Common Spadefoot Toads migration to wetlands.
- **Figure S3.** Plot showing the distribution and variation of data used in the model analysing the factors related to Common Spadefoot Toad phenological shift over time.
- **Figure S4.** Documentation of the predation of the Edible Frog on the Common Spadefoot Toad.
- **Table S1.** Parameters estimated by the model analysing the factors related to the number of Common Spadefoot Toads migrating to breeding wetlands considering minimum and maximum temperatures instead of mean temperature.
- **Table S2.** R^2 and AIC values of the model analysing the factors related to the number of Common Spadefoot Toads migrating to breeding wetlands considering mean, minimal and maximal temperature alternatively.
- **Table S3.** Parameters estimated by the model analysing the factors related to the number of Common Spadefoot Toads migrating to breeding wetlands divided by sex.
- **Table S4.** Pearson correlation matrix between independent variables related to the model analysing the factors related to the number of Common Spadefoot Toads migrating to breeding wetlands.

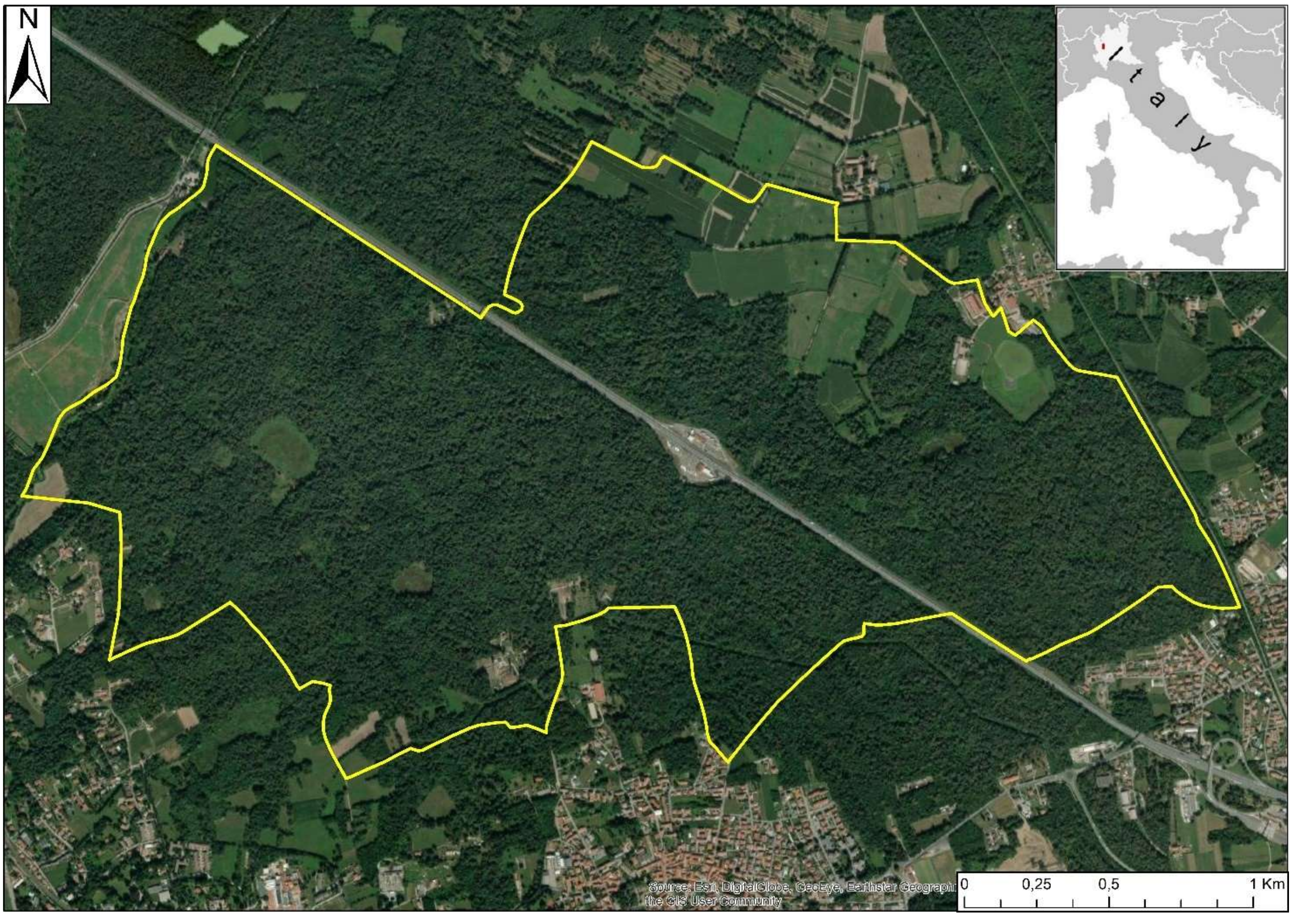

Figure S1. Location and satellite view of the Site of Community Importance "Paludi di Arsago". The yellow line represents site boundaries. The location of the wetlands has not been shown for conservation purposes. The map was generated by *AD* using the software ArcGis 10.3 (https://www.arcgis.com). Satellite Imagery credit: Esri, DigitalGlobe, GeoEye, Earthstar Geographics, CNES/Airbus DS, USDA, USGS, AeroGRID, IGN, and the GIS User Community.

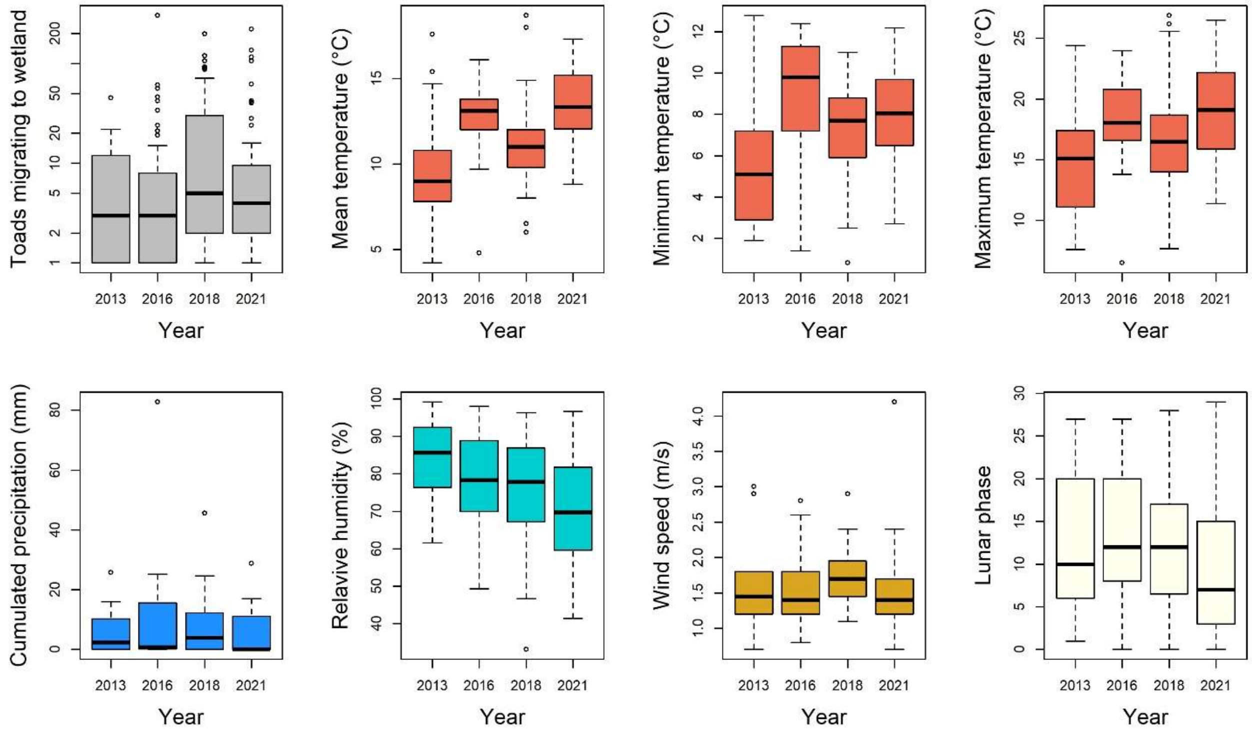

Figure S2. Plot showing the distribution and variation of data used in the model analysing the factors related to Common Spadefoot Toads migration to wetlands. All the values indicated in the boxplots refer to the period of monitoring of the Common Spadefoot Toad using pitfall traps (second half of March-May). From top-left to bottom-right: plot showing toad counts divided by year of monitoring; plot showing mean temperature values divided by year of monitoring; plot showing minimum temperature values divided by year of monitoring; plot showing maximum temperature values divided by year of monitoring; plot showing cumulated precipitation values divided by year of monitoring; plot showing relative humidity values divided by year of monitoring; plot showing wind speed values divided by year of monitoring; plot showing lunar phase values divided by year of monitoring. The length of the boxplot is represented by the difference between the 75th and 25th percentile, the black line represents the mean value, the dotted lines represent the upper and lower whiskers, the dots represent the outliers.

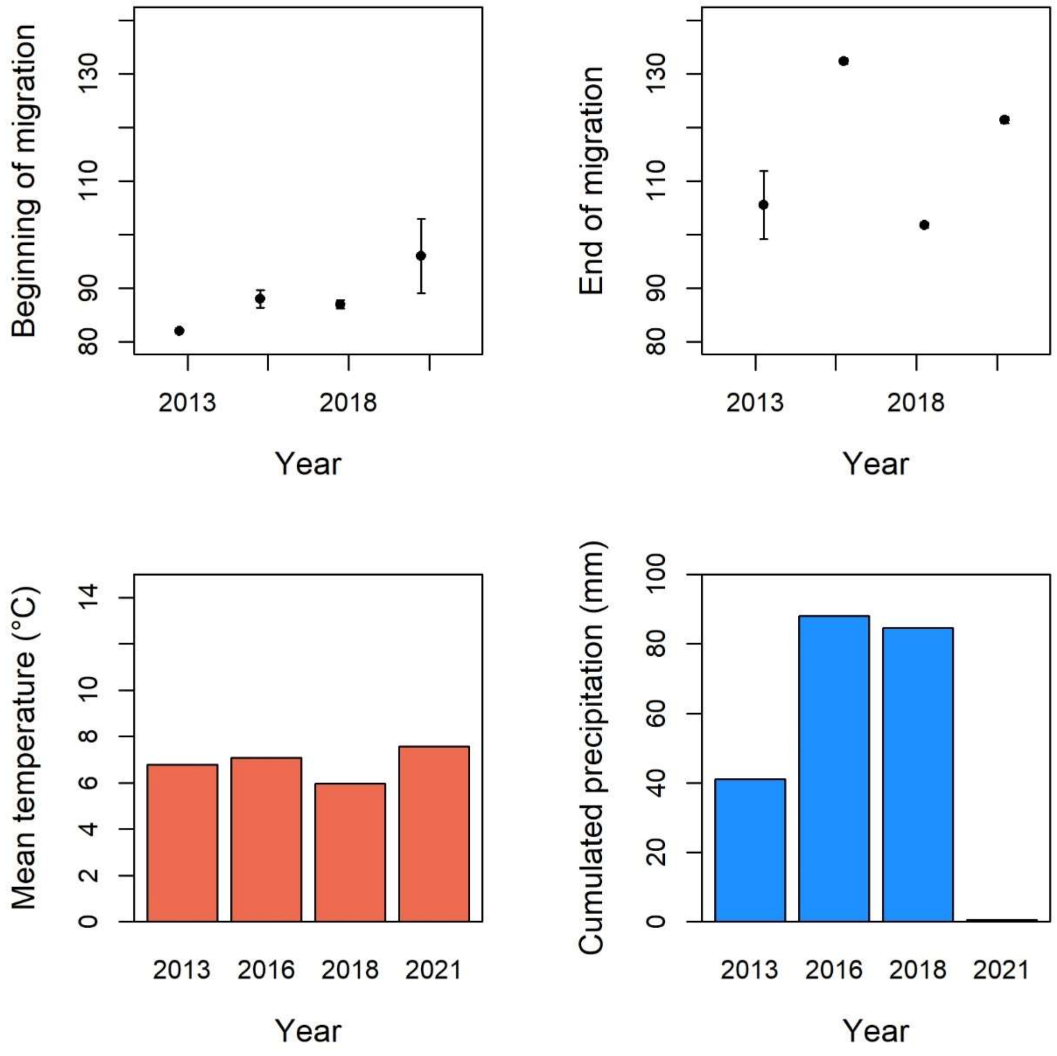

Figure S3. Plot showing the distribution and variation of data used in the model analysing the factors related to Common Spadefoot Toad phenological shift over time. All the values indicated in the bar plots refer to the period prior to monitoring activity of the Common Spadefoot Toad (first half of March). From top-left to bottom-right: plot showing changes in toad 10th percentile movement dates for each year of monitoring; plot showing changes in toad 90th percentile movement dates for each year of monitoring; plot showing changes in the mean temperature values in the first two weeks of March; plot showing changes in the cumulative precipitation values in the first two weeks of March. The black lines in the first two plot represent the 95% confidence interval.

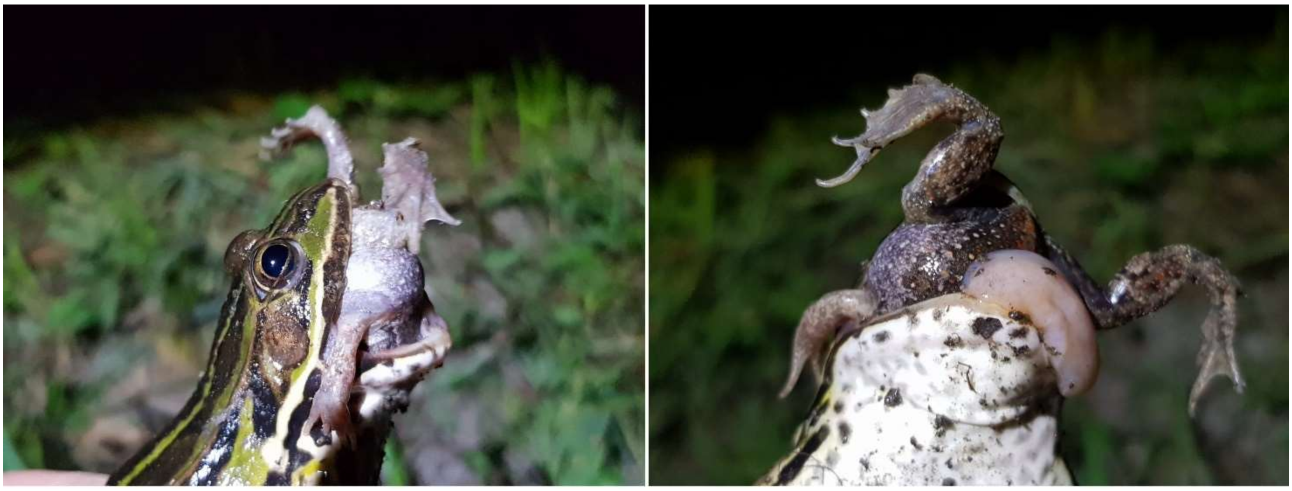

Figure S4. Photos showing an adult individual of Edible Frog eating a froglet of Common Spadefoot Toad. Pictures credits: PEB.

Table S1. Parameters estimated by the model analysing the factors related to the number of Common Spadefoot Toads migrating to breeding wetlands each day, considering minimum (a) and maximum (b) temperatures. Statistically significant parameters are highlighted in bold. se: standard error. The superscripts "^{2}" indicate quadratic terms.

a)

	Coefficient ± se	*z*-value	*p*-value
Intercept	1.52 ± 0.57	2.7	0.007
**Date**	**-0.46 ± 0.1**	**-4.51**	**< 0.001**
**Cumulative daily precipitation**	**0.62 ± 0.11**	**5.83**	**< 0.001**
Daily minimum temperature	0.09 ± 0.1	0.85	0.39
**Daily minimum temperature^2**	**-0.25 ± 0.07**	**-3.36**	**< 0.001**
**Relative humidity**	**0.41 ± 0.12**	**3.36**	**< 0.001**
Wind speed	-0.03 ± 0.09	-0.3	0.76
Moon phase	-0.02 ± 0.04	-0.5	0.62
Moon phase^2	-0.0009 ± 0.001	-0.7	0.48

b)

	Coefficient ± se	*z*-value	*p*-value
Intercept	1.21 ± 0.59	2.1	0.04
**Date**	**-0.48 ± 0.1**	**-4.75**	**< 0.001**
**Cumulative daily precipitation**	**0.67 ± 0.11**	**6.2**	**< 0.001**
Daily maximum temperature	-0.14 ± 0.13	-1.09	0.27
**Daily maximum temperature^2**	**-0.25 ± 0.06**	**-4.29**	**< 0.001**
Relative humidity	0.27 ± 0.15	1.74	0.08
Wind speed	-0.04 ± 0.09	-0.4	0.66
Moon phase	0.03 ± 0.04	0.76	0.45
Moon phase^2	-0.0007 ± 0.001	-0.6	0.54

Table S2. Conditional R^2, Marginal R^2, and AIC values of the model analysing the factors related to the number of Common Spadefoot Toads migrating to breeding wetlands each day, considering mean, minimum, or maximum temperature alternatively.

Model	Conditional R^2	Marginal R^2	AIC value
Mean temperature	0.793	0.339	1487.05
Maximum temperature	0.791	0.310	1498.64
Minimum temperature	0.773	0.328	1503.22

Table S3. Parameters estimated by the model analysing the factors related to the number of Common Spadefoot Toads migrating to breeding wetlands each day, divided by sex. Statistically significant factors are highlighted in bold. se: standard error. The superscripts "^{2}" indicate quadratic terms.

Model for males

	Coefficient ± se	z-value	*P*-value
Intercept	0.7 ± 0.62	1.11	0.27
**Date**	**-0.59 ± 0.12**	**-4.93**	**< 0.001**
**Cumulative daily precipitation**	**0.9 ± 0.1**	**8.65**	**< 0.001**
Daily mean temperature	-0.1 ± 0.12	- 0.79	0.43
**Daily mean temperature^2**	**-0.33 ± 0.07**	**-4.8**	**< 0.001**
**Relative humidity**	**0.39 ± 0.13**	**3.08**	**0.002**
Wind speed	-0.02 ± 0.09	-0.27	0.79
Moon phase	0.03 ± 0.04	0.8	0.42
Moon phase^2	-0.0009 ± 0.001	-0.65	0.51

Model for females

	Coefficient ± se	z-value	*P*-value
Intercept	1.02 ± 0.5	2.04	0.04
**Date**	**-0.4 ± 0.13**	**-3.16**	**0.001**
**Cumulative daily precipitation**	**0.82 ± 0.11**	**7.29**	**< 0.001**
Daily mean temperature	-0.07 ± 0.12	- 0.57	0.57
**Daily mean temperature^2**	**-0.43 ± 0.08**	**-5.35**	**< 0.001**
**Relative humidity**	**0.4 ± 0.13**	**3.08**	**0.002**
Wind speed	-0.02 ± 0.09	-0.27	0.78
Moon phase	0.03 ± 0.04	0.66	0.5
Moon phase^2	-0.001 ± 0.001	-0.77	0.44

Table S4. Pearson correlation matrix between independent variables related to the model analysing the factors related to the number of Common Spadefoot Toads migrating to breeding wetlands. Values in bold indicate correlations > |0.7|.

	**Date**	**T mean**	**T min**	**T max**	**Precipitation**	**Humidity**	**Moon phase**	**Wind Speed**
**Date**	1	0.45	0.59	0.27	0.37	0.13	0.17	-0.08
**T mean**	0.45	1	0.66	**0.9**	-0.12	-0.48	0.06	-0.13
**T min**	0.59	0.66	1	0.34	0.25	0.17	0.16	-0.19
**T max**	0.27	**0.9**	0.34	1	-0.32	-0.69	-0.02	-0.03
**Precipitation**	0.37	-0.12	0.25	-0.32	1	0.49	0.16	-0.02
**Humidity**	0.13	-0.48	0.17	-0.69	0.49	1	0.07	-0.32
**Moon phase**	0.17	0.06	0.16	-0.02	0.16	0.07	1	-0.01
**Wind speed**	-0.08	-0.32	-0.19	-0.03	-0.02	-0.32	-0.01	1
